# Selenium Hyperaccumulator Plants *Stanleya pinnata* and *Astragalus bisulcatus* Are Colonized by Se-Resistant, Se-Excluding Wasp and Beetle Seed Herbivores

**DOI:** 10.1371/journal.pone.0050516

**Published:** 2012-12-03

**Authors:** John L. Freeman, Matthew A. Marcus, Sirine C. Fakra, Jean Devonshire, Steve P. McGrath, Colin F. Quinn, Elizabeth A. H. Pilon-Smits

**Affiliations:** 1 Department of Biology, California State University Fresno, Fresno, California, United States of America; 2 Intrinsyx Technologies Corporation and Space Biosciences, N.A.S.A. Ames Research Center, Moffett Field, California, United States of America; 3 Lawrence Berkeley National Laboratory-Advanced Light Source, Berkeley, California, United States of America; 4 Rothamsted Research, Harpenden, Hertfordshire, United Kingdom; 5 Biology Department, Colorado State University, Fort Collins, Colorado, United States of America; Centro de Investigación y de Estudios Avanzados, Mexico

## Abstract

Selenium (Se) hyperaccumulator plants can concentrate the toxic element Se up to 1% of shoot (DW) which is known to protect hyperaccumulator plants from generalist herbivores. There is evidence for Se-resistant insect herbivores capable of feeding upon hyperaccumulators. In this study, resistance to Se was investigated in seed chalcids and seed beetles found consuming seeds inside pods of Se-hyperaccumulator species *Astragalus bisulcatus* and *Stanleya pinnata.* Selenium accumulation, localization and speciation were determined in seeds collected from hyperaccumulators in a seleniferous habitat and in seed herbivores. *Astragalus bisulcatus* seeds were consumed by seed beetle larvae (*Acanthoscelides fraterculus* Horn, Coleoptera: Bruchidae) and seed chalcid larvae (*Bruchophagus mexicanus*, Hymenoptera: Eurytomidae). *Stanleya pinnata* seeds were consumed by an unidentified seed chalcid larva. Micro X-ray absorption near-edge structure (µXANES) and micro-X-Ray Fluorescence mapping (µXRF) demonstrated Se was mostly organic C-Se-C forms in seeds of both hyperaccumulators, and *S. pinnata* seeds contained ∼24% elemental Se. Liquid chromatography–mass spectrometry of Se-compounds in *S. pinnata* seeds detected the C-Se-C compound seleno-cystathionine while previous studies of *A. bisulcatus* seeds detected the C-Se-C compounds methyl-selenocysteine and γ-glutamyl-methyl-selenocysteine. Micro-XRF and µXANES revealed Se ingested from hyperaccumulator seeds redistributed throughout seed herbivore tissues, and portions of seed C-Se-C were biotransformed into selenocysteine, selenocystine, selenodiglutathione, selenate and selenite. *Astragalus bisulcatus* seeds contained on average 5,750 µg Se g^−1^, however adult beetles and adult chalcid wasps emerging from *A. bisulcatus* seed pods contained 4–6 µg Se g^−1^. *Stanleya pinnata* seeds contained 1,329 µg Se g^−1^ on average; however chalcid wasp larvae and adults emerging from *S. pinnata* seed pods contained 9 and 47 µg Se g^−1^. The results suggest Se resistant seed herbivores exclude Se, greatly reducing tissue accumulation; this explains their ability to consume high-Se seeds without suffering toxicity, allowing them to occupy the unique niche offered by Se hyperaccumulator plants.

## Introduction

Selenium (Se) occurs naturally in certain soils, such as Cretaceous shale, at levels between 1 and 100 mg Se kg^−1^ [1], [2]. Selenium is biologically important because it is both an essential element to animals and toxic at high concentrations. Some plant species grow almost exclusively on seleniferous soils, and are characterized by extremely high Se concentrations in their tissues, reaching levels between 0.1 and 1.5% of dry weight (1,000–15,000 mg Se kg^−1^ DW). These plants, called Se hyperaccumulators, typically contain 100-fold higher Se levels than surrounding vegetation [1], [2].

Selenium hyperaccumulation defends plants via both deterrence and toxicity from a wide variety of herbivores (for a recent review see [3]). These include prairie dogs [4], phloem-sucking aphids [5], leaf chewing caterpillars [6], crickets and grasshoppers [7], and cell disrupting thrips and spider mites [8]. When given a choice, Se-sensitive herbivores avoid feeding on Se hyperaccumulator plants, and when forced to feed on high-Se leaves they suffer visible signs of Se toxicity and often die. The Se hyperaccumulator plants *Astragalus bisulcatus* (two-grooved milkvetch, Fabaceae) and *Stanleya pinnata* (Prince’s plume, Brassicaceae) harbored fewer arthropods in native seleniferous habitats compared to neighboring non-hyperaccumulator plants [9]. Selenium hyperaccumulator plants also cause devastating Se toxicity to livestock (e.g., cows, sheep and horses) [10].

**Figure 1:**
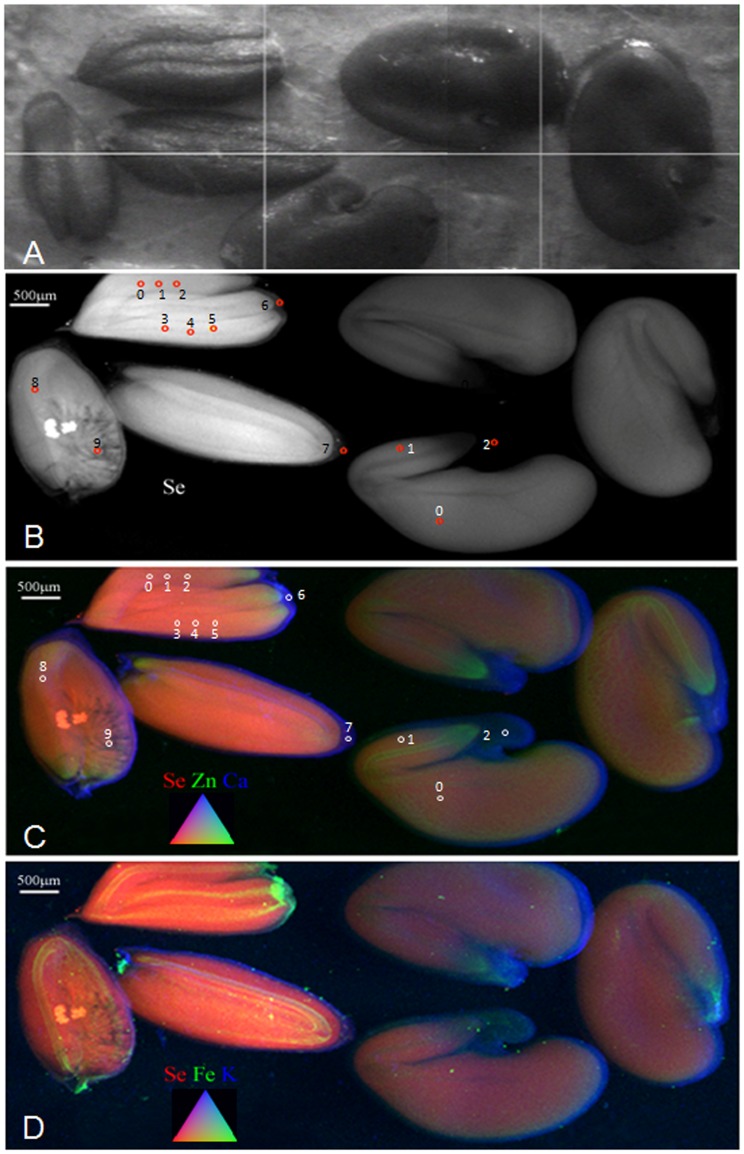
Localization of Se, Zn and Ca in seeds of Se hyperaccumulators *S. pinnata* and *A. biculcatus*. (a) Photograph of three seeds of *S. pinnata* (left) and three of *A. bisulcatus* (right) taken after synchrotron analysis. Note: at this time one of the *A. bisulcatus* seeds had shifted slightly toward the bottom left. (b) X-ray fluorescence (XRF) map showing Se distribution (in white) in the *S. pinnata* and *A. bisulcatus* seeds. (c) Tricolor-coded µXRF map of *S. pinnata* and *A. bisulcatus* seeds showing Se (in red), Zn (in green) and Ca (in blue). (d) Tricolor-coded µXRF map of *S. pinnata* and *A. bisulcatus* seeds showing Se (in red), Fe (in green) and K (in blue). The locations where XANES spectra were collected are indicated with numbered circles in panels B and C and results from XANES analyses are tabulated in Table 1.

Selenium can be toxic because plants inadvertently take up selenate (SeO_4_^2−^) via sulfate transporters, and assimilate it into seleno-amino acids via the sulfur assimilation pathway (for a review see [11]). Selenate is first reduced via selenite (SeO_3_^2−^) to selenide (Se^2−^), which is then incorporated into selenocysteine (SeCys) and selenomethionine (SeMet). One of the reasons Se is toxic is its similarity to sulfur (S), which can lead to the non-specific incorporation of Se into S-containing proteins and other metabolically important S compounds [12]. In addition to oxidative stress caused by the conjugation of glutathione to SeO_3_^2−^, the replacement of S in sulfhydryl groups or thiols (critical for disulfide bond formation) with Se can lead to a lack of normal protein conformation and result in structural malformation or loss of enzymatic activity [12]. Selenium hyperaccumulators circumvent Se toxicity by methylating SeCys via the enzyme SeCys methyl-transferase (SMT) and the resulting methyl-seleno-cysteine (MeSeCys) accumulates in a free pool because MeSeCys is not readily incorporated into proteins [13].

Micro-focused X-ray fluorescence (µXRF) mapping and Energy Dispersive X-ray Spectroscopy (EDS) demonstrated that Se hyperaccumulator plants preferentially hyperaccumulate Se in the periphery of leaves, in leaf hairs (called trichomes), or in vacuoles of leaf epidermal cells [14], [15]. Selenium X-ray absorption near-edge structure (µXANES) demonstrated that the majority (∼ 90%) of the Se in hyperaccumulator leaves consisted of organic carbon-selenium-carbon (C-Se-C) forms. Liquid chromatography mass spectroscopy (LCMS) only detected and quantified the C-Se-C compounds MeSeCys and γ-glutamyl-MeSeCys in a 1∶1 ratio in *A. bisulcatus* leaves, and MeSeCys and selenocystathionine (SeCyst) in a 4∶1 ratio in *S. pinnata* leaves [14]. Roots, florets and fruit of both hyperaccumulators harvested from the field also contained mainly organic C-Se-C forms [16], [17].

Although they deter many Se-sensitive herbivores, there is mounting evidence that Se hyperaccumulator plants may provide a niche occupied by Se-tolerant herbivores. For example a population of Se-tolerant *Plutellidae* closely resembling the diamondback moth (*Plutella xylostella*), was discovered in a seleniferous area near Fort Collins, CO, U.S.A. and was shown in laboratory tests not to avoid plants containing hyperaccumulated Se, and to readily oviposit and voraciously feed, on *S. pinnata* leaves that contained more than 2,000 µg Se g^−1^ DW, without suffering Se-toxicity [18]. In contrast, a population of diamondback moth originally collected from a non-seleniferous area in the Eastern U.S.A. preferred to oviposit and feed on *S. pinnata* plants containing trace Se concentrations, and suffered Se-toxicity and quickly died when fed Se rich leaves [18]. Potentially explaining the biochemical mechanism for the observed difference in Se tolerance, the Se-tolerant moth was found to accumulate MeSeCys, similar to its host plant *S. pinnata*, while the Se-sensitive population accumulated the de-methylated form SeCys and showed deterioration of multiple internal organs [18]. In the same study, the Se-tolerant *Stanleyii* moth larvae were found to be actively parasitized by a Se-tolerant microgastrine wasp, *Diadegma insulare* (Braconidae), which also accumulated MeSeCys. Thus, the co-evolution of Se hyperaccumulator plants, Se-tolerant herbivores, and Se-tolerant predators may represent a unique portal for Se to move up into higher trophic levels.

**Figure 2:**
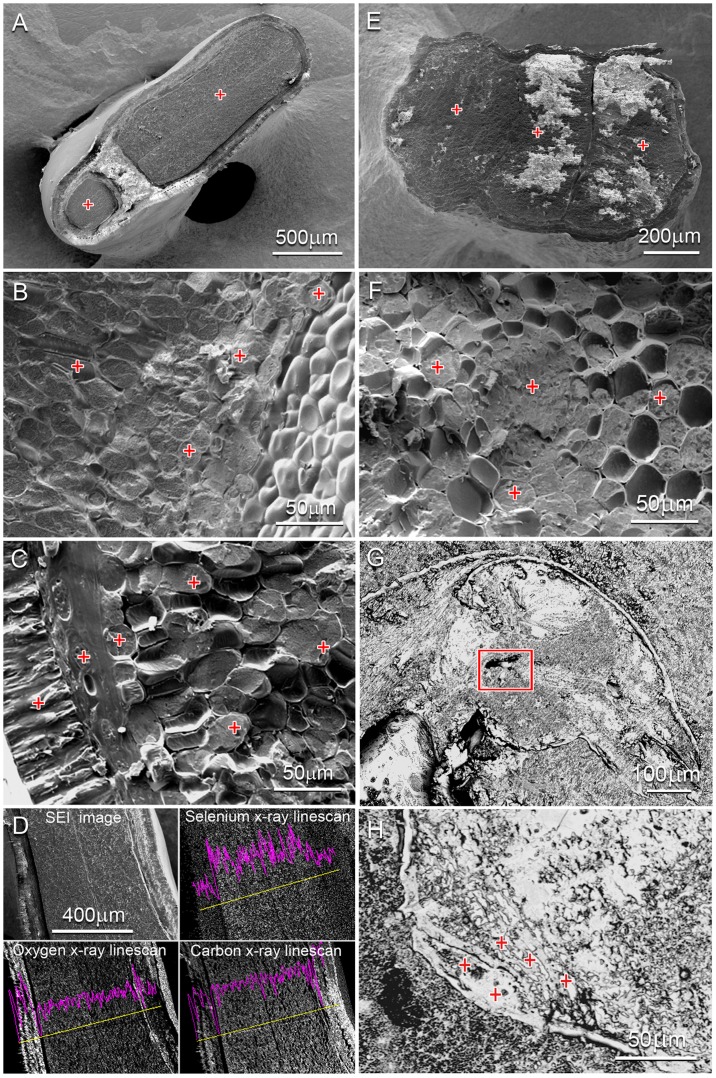
Scanning electron micrographs of Se hyperaccumulator seeds and cryo-fractured seed herbivorous beetle. Red crosses indicate acquisition points giving positive Se signals using energy dispersive x-ray spectrometry (EDS). Images A & D are *Astragalus bisulcatus* seeds, air dried and cross-sectioned. The x-ray linescan acquired (D) shows that Se levels drop at both seed-coat (testa) edges. Images B & C are *Astragalus bisulcatus* seeds frozen and cryo-fractured across (B) the endosperm region and (C) the testa, which is visible in the lower left corner of the image. Images E &F are *Stanleya pinnata* seeds, (E) air-dried and cross-sectioned and (F) frozen and cryo-fractured across the endosperm region. Images G & H are SEM back-scatter images of (G) frozen, cryo-fractured lateral view of the seed beetle head. The area of the insect’s mouth outlined in red was negative for Se. Image (H) is a ventral view of the bruchid beetle’s lower abdomen, frozen and cryo-fractured. Spectra included in Material S1.

**Table 1:** Chemical forms of Se found in seeds of *S. pinnata* and *A. bisulcatus*.

	SS (x10^−4^)	SeO_3_^2−^	Se(GSH)_2_	C-Se-C	Se^0^
*S. pinnata*					
0, 1, 2 embryo, root	3.3	3%	nd	77%	19%
3, 4, 5 embryo, cotyledon	4.1	3%	nd	73%	24%
6, 7 seed coat	1.8	5%	nd	77%	19%
8, 9 embryo, herbivore damage	3.1	nd	nd	100%	nd
*A. bisulcatus*					
0 embryo, cotyledon	3.7	3%	nd	97%	nd
1 embryo, root	4.0	3%	nd	96%	nd
2 seed coat	9.8	8%	28%	63%	nd

Results from least-squares linear combination fitting of each samples XANES spectra in comparison to standard selenium compounds.

The regions where the spectra were collected are indicated in Figure 1.

SeO_3_^2−^: selenite; Se(GSH)_2_: seleno-diglutathione, C-Se-C: methyl-selenocysteine, seleno-methionine or seleno-cystathionine.

Se^0^: red or gray elemental Se. SS: normal sum of squares (quality of fit; 0 = perfect fit); nd: compound not detectable. Additional standard compounds included in the fit but not detected in any location were selenate, seleno-cystine and seleno-cysteine. Note: fractions do not always add up to exactly 100% because the margin of error can be up to10%.

Seeds contain the highest Se concentrations of all the organs of Se hyperaccumulator plants [14], [16], [19]. Selenium is thought to be actively transported from ageing leaves to reproductive organs, and to be highly concentrated in seeds. The form(s) of Se remobilized to seeds of hyperaccumulator plants may be organic, since *A. bisulcatus* seeds have been reported to accumulate both MeSeCys and γ-glutamyl-MeSeCys [20]. It may enhance the reproductive success of a Se hyperaccumulator plant if Se is concentrated in the seed, where it can protect both seed and the newly germinating seedlings from herbivory or pathogen infection. For example, *Acanthoscelides mixtus* and *Acanthoscelides pullus* seed beetle larvae, hatched from eggs oviposited by adults, were found feeding on seeds inside seedpods of the Se hyperaccumulator plant *A. praelongus*, but intriguingly the adults never successfully emerged. However, these same seed beetle larvae successfully completed their lifecycle and emerged from seed pods after consuming seeds of several non-accumulator *Astragalus* species that do not contain high concentrations of Se [21]. A third seed beetle species, *Acanthoscelides aureoles*, was found to consume seeds of both Se hyperaccumulator and non-accumulator plant species with equal success. This finding led the authors to hypothesize that some seed beetles may have co-evolved with Se hyperaccumulators and evolved Se resistance. Trelease and Trelease [22] also reported the presence of a seed beetle consuming seeds of Se hyperaccumulating *A. bisulcatus* containing 1,475 µg Se g^−1^, which they identified as *Acanthoscelides fraterculus* (Horn), a species very closely related to *A. aureoles*. Furthermore, Trelease and Trelease observed large numbers of seed chalcids, small wasp-like hymenopteran insects eating *A. bisulcatus* seeds, which they identified as *Bruchophagus mexicanus* (Ashmead) [22]. Lavigne and Littlefield [23], in their annotated list of insects associated with *Astragalus* species, report that the seed beetle *A. fraterculus* has been found in seed pods of at least three Se hyperaccumulator plant species: *A. bisulcatus*, *A. racemosus*, and *A. pectinatus*. Lavigne and Littlefield mention *Bruchophagus mexicanus* in seed pods of the closely related, often neighboring Se hyperaccumulator *A. racemosus*, as well as in several non-accumulating *Astragalus* species [23].

**Figure 3:**
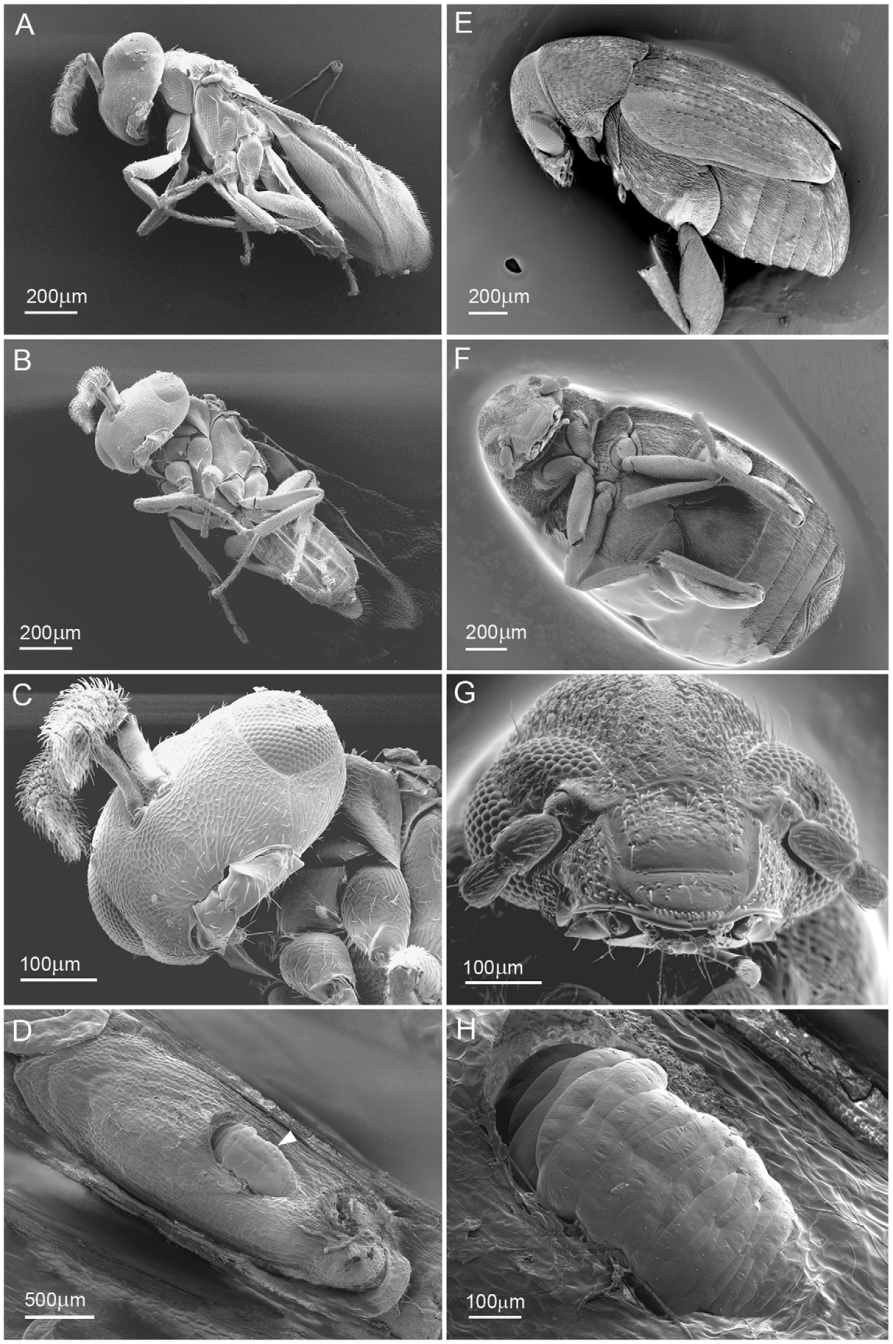
Scanning Electron Micrographs of seed chalcid wasp, seed chalcid wasp larva (in situ *Stanleya pinnata* seed) and seed beetle. Images A–C are views of the chalcid wasp, areas around the mouth, thorax, legs and abdomen were targeted for Se using EDS. On these external surfaces only very low levels of Se were detected from upper leg segment spines (more detail is provided in Material S1). The seed chalcid wasp larva in image D (white arrow head) & H at higher magnification gave low positive Se signals around a spiracle and also on some bristles/spines seen on the external surface (Material S1). Images E – G are views of the seed beetle, which was also targeted for Se around the mouth, legs and abdomen using EDS, with no positive signals detected.

**Figure 4:**
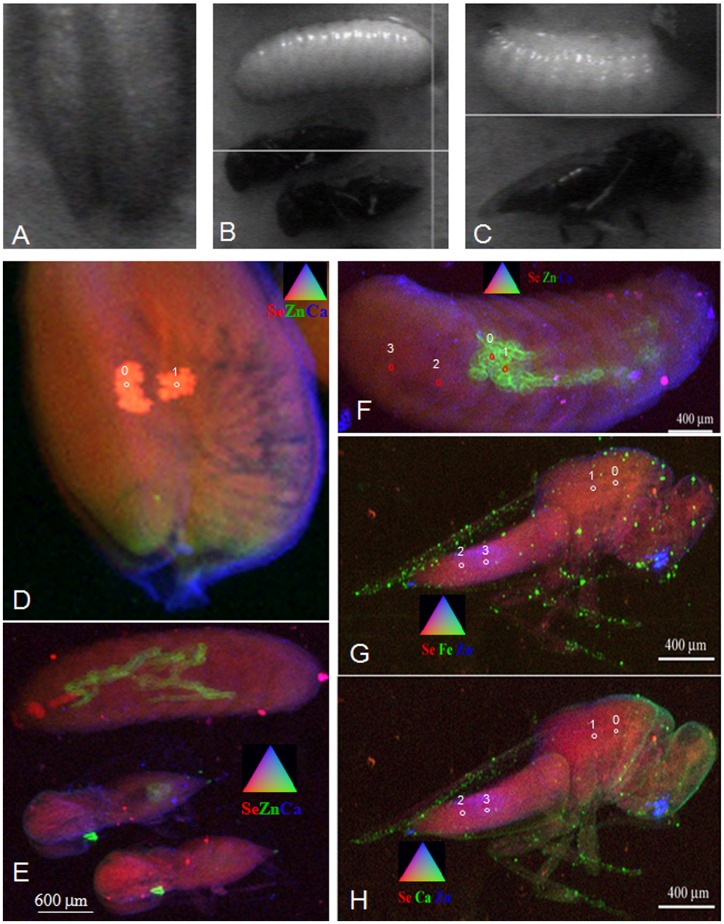
Localization of selected elements in seeds of Se hyperaccumulator *S. pinnata* and a seed herbivorous chalcid wasp. Photographs of (a) *S. pinnata* seed, (b) seed chalcid wasp larvae and adults associated with (c) *S. pinnata* seeds. (d) Tricolor-coded µXRF map of the *S. pinnata* seed showing Se (in red), Zn (in green) and Ca (in blue). The Se-rich areas are frass from the seed chalcid wasp larvae (e) Tricolor-coded µXRF map of the seed chalcid wasp larva and adults showing Se (in red), Zn (in green) and Ca (in blue). (f) Tricolor-coded µXRF map of the seed chalcid wasp larva showing Se (in red), Zn (in green) and Ca (in blue). (g) Tricolor-coded µXRF map of the seed chalcid wasp adult showing Se (in red), Fe (in green) and Zn (in blue). (h) Tricolor-coded µXRF map of the seed chalcid wasp adult showing Se (in red), Ca (in green) and Zn (in blue). The locations where XANES spectra were collected are indicated with numbered circles and results from XANES analyses are displayed in Table 2.

In order to investigate Se resistance observed in the insect herbivores of Se hyperaccumulator seeds at the molecular level we mapped the distribution of Se and analyzed the forms of Se accumulated in seeds of *A. bisulcatus* and *S. pinnata,* and three associated insect herbivores feeding on these plants. The results provide insight into the Se resistance mechanism, at a molecular level, of the seed herbivores and help assess the potential for them to bio-transfer Se to higher trophic levels in seleniferous ecosystems.

## Materials and Methods

### Collection of Biological Material

*Astragalus bisulcatus* (Hook.) A. Gray and *S. pinnata* (Pursh) Britton seeds were collected in the summer (June-July) at Pine Ridge Natural Area, a seleniferous site west of Fort Collins, CO, USA that has been described before [9], [19]. Seeds that were used for elemental analysis were dried, ground in a mortar and pestle, acid-digested and analyzed for total Se and S via inductively coupled plasma atomic emission spectrometry as described below (n = 22 for *S. pinnata* and n = 6 for *A. bisulcatus*). Seeds for XAS analyses and LCMS were flash-frozen in liquid nitrogen and kept at −80°C until analyzed as described below. Seed pods to be used for collection of herbivores were harvested from Pine Ridge and either immediately dissected and any larvae were extracted using forceps, or whole intact seed pods were placed in aquaria sealed with a fine nylon mesh in a 25°C growth room under 12 h photoperiod until adult herbivores emerged, at which point they were captured. Seeds and live herbivores were then chilled to 4°C and packaged for transport to Rothamsted Research UK for EDS analysis as described below. A subsample of larvae and adults were flash frozen alive in liquid nitrogen and shipped to the Lawrence Berkeley National Laboratory Advanced Light Source for µXRF and µXANES. The three seed herbivores were deposited in the C.P. Gillette Museum of Arthropod Diversity at Colorado State University.

**Figure 5:**
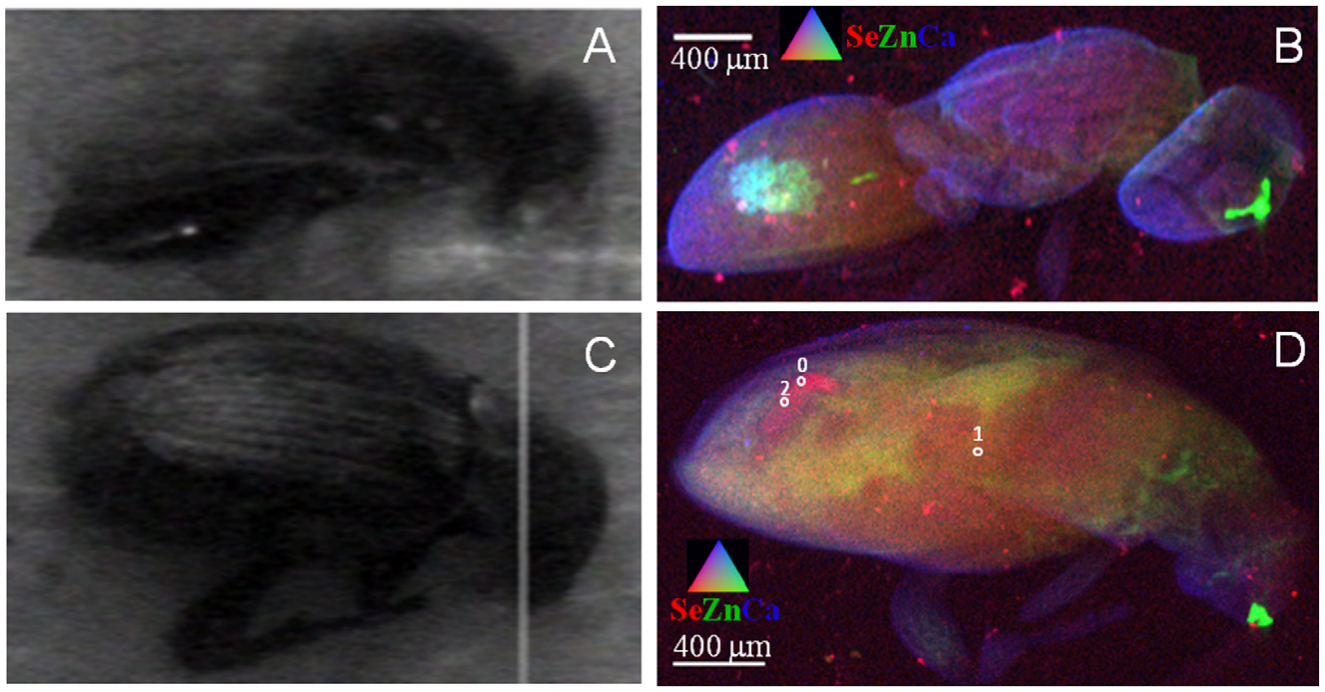
Localization of Se, Zn and Ca in two seed herbivores of Se hyperaccumulator *A. bisulcatus*, a seed herbivorous chalcid wasp and a seed beetle. (a) Photograph of a chalcid wasp that emerged from *A. bisulcatus* seed. (b) Tricolor-coded µXRF map of the chalcid wasp showing Se (in red), Zn (in green) and Ca (in blue). (c) Photograph of a seed beetle that emerged from seed of *A. bisulcatus*. (d) Tricolor-coded µXRF map of the seed beetle showing Se (in red), Zn (in green) and Ca (in blue). The locations where XANES spectra were collected are indicated with numbered circles and results from XANES analyses are displayed in Table 2.

**Table 2:** Chemical forms of Se found in herbivore insects, and frass obtained from least squares linear combination fitting of each samples XANES spectra in comparison to standard selenium compounds.

	SS (x10^−4^)	SeO_4_^2−^	SeO_3_^2−^	Se(GSH)_2_	SeCysteine	SeCystine	C-Se-C
*Stanleya pinnata* Seed Insects							
4D (0, 1) Seed wasp larva frass inside seed	11	2%	nd	nd	nd	nd	96%
4F (0, 1) Seed wasp larva, gut	19	nd	16%	57%	nd	29%	nd
4F (2, 3) Seed wasp larva, tissue	5.2	nd	10%	43%	nd	nd	46%
4GH (0, 1) Seed wasp adult, thorax	4.8	nd	9%	21%	nd	nd	70%
4GH (2, 3) Seed wasp adult, abdomen	6.3	nd	8%	29%	25%	8%	28%
*Astragalus bisulcatus* Seed Insect							
5D 0, 1, 2 Seed beetle adult	7.0	nd	14%	34%	nd	53%	nd

XANES spectra were obtained from organisms at locations shown in Figures 4 and 5. SeO_4_^2−^: selenate SeO_3_^2−^: selenite; Se(GSH)_2_: seleno-diglutathione; C-Se-C: methyl-selenocysteine, seleno-methionine or seleno-cystathionine. SS: normal sum of squares (quality of fit; 0 = perfect); nd: compound not detectable.

No elemental Se^0^ was found in these samples. Note: fractions do not always add up to exactly 100% because the margin of error can be up to10%.

### Measurement of Total Se and S Concentrations, and Identification of Non-protein Organic Selenocompounds

Inductively coupled plasma atomic emission spectrometry (ICP-AES) was used to determine the concentrations of total Se and S [24]. The whole biological material was rinsed with distilled water and dried for 48 h at 45°C. Samples were then finely ground using a mortar and pestle and digested in nitric acid as described by Zarcinas *et al.* [25]. Liquid Chromatography Mass Spectrometry (LC-MS) was used to determine the chemical speciation of the Se-compounds in *S. pinnata* seeds, as described by Freeman *et al*. [14], [18].

### X-ray Microprobe Analyses

Selenium distribution and speciation were investigated using µXRF and µXANES, respectively, as described by Freeman *et al.* [14], [18]. Three seeds were analyzed for *S. pinnata* and three for *A. bisulcatus*. Fresh, intact biological samples were flash-frozen in liquid nitrogen and shipped on dry ice to beamline 10.3.2 at the Lawrence Berkeley National Laboratory Advanced Light Source (LBNL-ALS), Berkeley, CA for microprobe analyses [26]. Frozen samples were placed on a −27°C Peltier stage to reduce potential radiation damage. Micro-XRF elemental maps were recorded at 13 keV. The chemical forms of Se in particular areas of interest were analyzed using Se K-edge XANES. Micro-XRF maps and µXANES spectra were recorded with a 7 element Ge solid state detector (Canberra, ON, Canada). Spectra were deadtime corrected, pre-edge background subtracted, and post-edge normalized using standard procedures [27]. Red selenium (white line energy set at 12660 eV) was used to calibrate the spectra. Least-square linear combination (LSQ) fitting of Se XANES spectra was performed in the 12630–12850 eV range, using a spectral library of standard Se compounds. The error on the fit percentages of Se species was estimated at ±10%. Standards used were: Na_2_SeO_4_, Na_2_SeO_3_, selenocystine, selenomethionine purchased from Sigma-Aldrich (St Louis, MO, USA), methylselenocysteine, γ–glutamyl-methylselenocysteine, and selenodiglutathione were purchased from PharmaSe (Austin, TX, USA). Selenocysteine was obtained by reducing selenocystine overnight at 25°C in 100 mM sodium borohydride at a 1∶1 molar ratio. Gray and red elemental Se^(0)^ standards were provided by Amy Ryser and Dan Strawn at LBNL-ALS. Data processing and analyses were performed using custom LabVIEW (National Instruments, Austin, TX, USA) software programs available at the beam line.

### Energy-dispersive X-ray Spectroscopy

Energy Dispersive Spectrometry (EDS) and was used to investigate the localization of Se in cryo-fractured and cryo-planed samples. For cryo-fracturing the seeds were mounted on a cryo stub using OCT compound (Sakura-Netherlands) and plunge-frozen in pre-slushed liquid nitrogen (LN_2_). The seeds were transferred under vacuum to the GATAN Alto 2100 cryo chamber (Gatan UK) with temperature maintained at −180°C. Here they were fractured using the cold blade mounted in the prep chamber and any contaminating ice was removed through sublimation by raising the stage temperature to −95°C for 1 minute. The heater was then turned off and the stage temperature was allowed to recover to −160°C. For cryo-planing the insect samples were embedded in OCT compound (Sakura-Netherlands), plunged into liquid nitrogen and then mounted in the cryostat LeicaCM1850 where the surface was planed using a steel blade at −30°C. This technique is easy to stop once the plane of interest has been reached and the sample is then transferred under liquid nitrogen to the prep chamber attached to the microscope for etching and coating. The specimens were then sputter coated with Au for 60 sec, (approximate thickness 10 nanometers) and transferred to the JSM LV6360 (Jeol UK) scanning electron microscope stage for examination. The microscope stage was maintained at −160°C and after imaging the parameters were set for EDS analysis using the OXFORD INCA 2000 microanalysis system (Oxford Instruments, UK). An accelerating voltage of 5000V (Se Lα line detected) instead of the higher voltage needed to see the Se K line was chosen for the analysis, as it is less damaging to fully hydrated frozen biological material, reduces movement of labile elements and avoids interference from other elements such as the Au coating. Air-dried samples were also prepared and examined on the dry stage at room temperature for selenium.

**Table 3:** Total Se and S concentrations, and Se/S concentration ratios in seeds and seed-herbivores of *S. pinnata* and *A. bisulcatus*, as determined by ICP-AES.

	Se (mg kg ^−1^ DW)	S (mg kg ^−1^ DW)	Se/S
*S. pinnata* seeds (n = 22)	1,329±244	12,051±420	0.110
*S. pinnata* seed wasp larva (n = 6)	9±2	1,574±73	0.006
*S pinnata* seed wasp adult (n = 4)	47±6	5,048±2150	0.009
*A. bisulcatus* seeds (n = 6)	5,750±754	11,837±1486	0.486
*A. bisulcatus* seed wasp adult (n = 3)	6±1	2,815±347	0.002
*A. bisulcatus* seed beetle adult (n = 3)	4±1	2,241±101	0.002

Data shown is the mean and standard error. Replicate samples were collected from one or more individuals.

## Results

Micro-XRF mapping shows that in seeds of both *S. pinnata* and *A. bisulcatus* Se was mainly concentrated throughout the embryo and a much lower level was present in the seed coat (Fig. 1B, 1C). Within the embryo, Se was fairly evenly distributed, although the Se signal appears somewhat less strong in the seed embryo vascular tissue. Zinc (Zn) and iron (Fe) were also present in the embryo but excluded from the seed coat; they were strongly concentrated in the vascular tissue, particularly at the root tip (Fig. 1C, 1D). One of the *S. pinnata* seeds showed mandible scrape scars indicative of larval herbivory, part of the seed embryo was eaten (Fig. 1B) and Se rich larval frass (i.e. feces) was found in the seed pod (Fig. 1B). Micro-XANES analysis revealed that the majority of Se (∼63–100%) in both the *S. pinnata* and *A. bisulcatus* seeds was in C-Se-C forms, such as SeMet, MeSeCys, γ-glutamyl-MeSeCys or seleno-cystathionine (Table 1). In addition to C-Se-C forms, the *S. pinnata* seeds contained elemental Se (19–24%) and a trace of selenite (up to 5%), and the *A. bisulcatus* seeds also contained a trace of selenite (3–8%) (Table 1). Further analyses by LC-MS detected and identified seleno-cystathionine as the only detectable C-Se-C form in *S. pinnata* seeds (Fig. S1). In *A. bisulcatus* seeds the form of Se was reported in the literature to be MeSeCys and γ-glutamyl-MeSeCys [28], which is in agreement with our C-Se-C XANES data. EDS analysis indicated the presence of Se in cells of the fractured surfaces of both seed types (Fig. 2). In Figure 2D the Se X-ray line-scan across the fractured surface of *A. bisulcatus* demonstrates how the concentration starts off low at the seed coat (testa) edge, increases across the endosperm region and then drops down again at the other seed coat edge. Fractures across the air-dried whole seeds of *A. bisulcatus* and *S. pinnata* (Fig. 2A and 2E) show acquisition points across the endosperm all positive for Se at varying levels. The cryo-fractured fully hydrated seeds at higher magnification (Fig. 2B, 2C and 2F) all gave positive readings for Se including one from the seed coat layers (spectra available in Material S1).

Seeds of *S. pinnata* and *A. bisulcatus* that had been collected from a seleniferous field site were dissected and found to contain three different herbivorous insect species: the *S. pinnata* seeds contained an unknown seed chalcid wasp, and the *A. bisulcatus* seeds harbored a larger seed chalcid wasp as well as a seed beetle (Figs. 3, 4 and 5). Based on morphology we tentatively identified the *A. bisulcatus* seed beetle as *Acanthoscelides fraterculus* Horn, Coleoptera: Bruchidae (Figs. 3, 5) and the seed chalcid wasp resembled *Bruchophagus mexicanus*, Hymenoptera: Eurytomidae) (Figs. 3, 4). Energy-dispersive X-ray spectroscopy (EDS) detected very little Se in the herbivore tissues, except for a very weak signal in the mid-abdomen and gut of the bruchid beetle and the posterior abdomen region of the cryo-fractured samples (Figs. 2, 4). The seed beetle mouth region indicated by the red rectangle in Figure 2G was surprisingly negative for Se even though Se was detected inside the beetle’s body. External surfaces of the seed chalcid wasp, seed chalcid wasp larva and the seed beetle were also targeted for Se analysis using EDS. Very low levels of Se were detected on upper leg segment spines of the chalchid wasp and around the spiracle and some bristles/spines on the seed chalcid larva.

Micro-XRF mapping of the larvae and adult seed chalcid wasp that emerged from the *S. pinnata* seed pods demonstrated that Se was present throughout all tissues in both stages (Fig. 4E–H). In the seed chalcid wasp larva Se was uniformly distributed (Fig. 4F) and in the seed chalcid wasp adult the Se concentration was elevated in the thorax and abdomen, and lower in the wings and exoskeleton (Fig. 4G, 4H). Iron was concentrated in discrete locations along the adult’s exoskeleton and on wings; some of these Fe “hot spots” also contained Ca (Fig. 4G, 4H). Zinc was concentrated in the intestine of the larva and in mouth parts (mandibles) of the adults (Fig. 4E–H).

XANES analysis (Table 2) showed that the high-Se frass deposited by the seed chalcid wasp larvae in the *S. pinnata* seed contained almost exclusively (96%) C-Se-C forms, the same forms in the seed embryo (Table 2, *S. pinnata* spectra 0 and 1). On the other hand, the chalcid wasp larva that emerged from *S. pinnata* seeds contained only 46% C-Se-C forms in tissues and no C-Se-C forms were detected in the midgut (Table 2). A large fraction (43–57%) of the Se in the larva was Se-diglutathione (Se(GSH)_2_) and 10–16% was selenite (Table 2). The Se XANES spectrum obtained from the midgut also indicated the presence of SeCystine (29%), however this compound did not have a very good fit likely due to the relatively low Se concentrations. In the adult chalcid wasp the Se speciation varied somewhat between thorax and abdomen (Table 2). The Se XANES spectrum collected at the thorax contained 70% C-Se-C, while the abdominal spectrum had only 28% C-Se-C; furthermore, the abdomen contained SeCys (25%) and trace levels of SeCystine (8%) while these compounds were not detected in the thorax. Both thorax and abdomen also contained fairly large fractions of Se(GSH)_2_ (21–29%) and trace levels of selenite (8–9%).

Micro-XRF mapping of the seed chalcid wasp and seed beetle adults after emerging from the *A. bisulcatus* seed pods demonstrated that Se was present throughout the insects (Fig. 5). In the seed chalcid wasp, the Se did not appear to be concentrated in any particular area, but in the beetle Se was apparently accumulated in the hindgut (Fig. 5D). Zinc was concentrated in the mandibles of both animals, and in the intestine (Fig. 5B, 5D). Unfortunately, the Se signal from the seed chalcid wasp adult was too low to obtain XANES spectra. The Se in the seed beetle adult, however, was concentrated enough for spectra to be obtained in three locations, which all gave similar results and demonstrated that the forms of Se were SeCystine (53%), Se(GSH)_2_ (34%) and selenite (14%) (Table 2).

To further gain a molecular understanding into how these seed insect herbivores can feed on seeds containing such extremely high levels of Se, ICP-AES analysis was done on seeds and herbivores in order to quantify total Se and S levels and then compare and contrast them to one another. Sulfur was included in the analysis because of its biochemical similarity to Se. Nineteen *S. pinnata* seed samples were analyzed that were collected from at least one plant each. The seeds contained on average 1,329 mg Se kg^−1^, with a range between 261 and 3,293 mg Se kg^−1^ (Table 3). This was in stark contrast to the Se levels in the seed chalcid wasp that had fed on *S. pinnata* seeds. For example, when compared to the *S. pinnata* hyperaccumulator plant seeds, the seed chalcid wasp larva and adult contained on average 148- and 28- fold lower Se concentrations at 9 and 47 mg Se kg^−1^ respectively (Table 3). Similarly, for *A. bisulcatus* seeds and its seed insect herbivores a vast difference in Se concentration was observed. Intriguingly, the two herbivore species contained Se concentrations that were three orders of magnitude lower than the Se concentrations in the seeds they had just fed on (Table 3). *Astragalus bisulcatus* seeds showed a range in Se concentration between 3,918 and 8,349 mg Se kg^−1^, while the herbivores contained less than 10 mg Se kg^−1^. These results support the above findings that Se is not accumulating in tissues and based on the forms present in seed insect herbivores, Se must be actively excluded. All seed insect herbivores also contained lower S concentrations than Se hyperaccumulator plant seeds, but the difference in S concentration between seed and seed insect herbivores was much smaller (2- to 7.5- fold, Table 3). As a result, the Se/S ratio in the herbivores was 12–18 times lower in the *S. pinnata* herbivore than in *S. pinnata* seeds, and 243-fold lower in the two *A. bisulcatus* seed herbivores compared to the *A. bisulcatus* seeds (Table 3, right column).

## Discussion

In this study the accumulation, distribution and chemical forms of Se were analyzed in seeds of two Se hyperaccumulators, *A. bisulcatus* and *S. pinnata*, as well as in three associated seed herbivore species. Both plant species hyperaccumulated Se to extraordinarily high concentrations throughout the seed embryo and endosperm, however, little Se was detected in the seed coat, as judged from µXRF and EDS analyses. XANES demonstrated that the forms of Se in seeds were C-Se-C, identified as Se-cystathionine in *S. pinnata* seeds, and previously reported by Nigam and McConnell [20] to be MeSeCys and γ-Glu-MeSeCys in *A. bisulcatus* seeds. This study is the first to investigate the form of Se in *S. pinnata* seeds and the presence of only one form, seleno-cystathionine, is remarkable based on previous research showing multiple forms of Se in other *S. pinnata* tissues and organs. Leaves of *S. pinnata* were found earlier to contain a substantial fraction of Se as seleno-cystathionine, but the majority of Se in leaves was MeSeCys [14]. Thus, assuming the speciation data from independent studies using plant material collected at different time points can be compared; Se speciation appears to be different in seeds than in leaves of *S. pinnata*. It is possible that selenocystathionine is selectively translocated from leaves and directed into seeds. In *A. bisulcatus*, however, leaves and seeds both contain MeSeCys and γ-glutamyl-MeSeCys [14], [20]. Distinctively, *S. pinnata* seeds also contained up to 24% elemental Se^(0)^, which has not been reported in seeds but is often associated with microbial activity [29], [30]. Recently, elemental Se^(0)^ was discovered in roots of *S. pinnata* and *A. bisulcatus* when growing in the native ecosystem on seleniferous soil, but this was not observed in the greenhouse [17]. At this point it is not clear whether these hyperaccumulators can make elemental Se^(0)^ themselves or whether it results from the action of endophytic or closely associated microbes. Sun *et al*. [31] reported for rice grains that SeMet was the major Se species (83% of total Se), and that the grains also contained relatively small fractions of MeSeCys and SeCys. Similar to rice grains, wheat kernels accumulated mainly (72–85%) SeMet [32]. The Se hyperaccumulated in these two species seeds appeared to be mainly organic as well. The finding that the forms of organic Se in seeds of hyperaccumulators can be quite different between these plant species from two different families is of significance, since high concentrations of different selenocompounds have varying levels of Se toxicity, but also a range of antioxidant and anticarcinogenic efficacies when consumed by animals or humans at much lower, nutritionally ideal concentrations [33]. Another difference between rice and *S. pinnata* or *A. bisulcatus* was that the Se concentration in rice bran (the seed coat) was 2-fold higher than that in the corresponding polished rice [31], while in the hyperaccumulator plants Se was present mostly in the seed embryos as opposed to the much lower concentrations found in seed coats.

While not the focus of this study, it was an interesting observation that each of the three herbivores showed substantial accumulation of Zn in their mouth parts. This was also observed in the diamondback moth in earlier µ-XRF studies [18]. As mentioned by Schofield *et al*. [34], Zn and other heavy metals are often found in the mouth parts of arthropods. In the mandibles of leaf-cutter ants Zn accumulated with age, up to 16% of DW. The metals likely function to provide strength to the mouth parts, as mandible hardness was found to be correlated with Zn content [34].

The exact Se levels in the seeds these particular seed herbivores emerged from is not known, but based on the range in Se concentration in seeds collected from this area it appears contained Se levels several orders of magnitude lower than those in the seeds that they fed on (Table 3). This strongly suggests that all three herbivores are Se excluders.Further evidence for this hypothesis is that the frass of the seed chalcid wasp larvae in the *S. pinnata* seed was much more highly concentrated in Se when compared to the Se in the rest of the seed (Fig. 1). The mechanism of Se exclusion may be fairly Se-specific, although due to the chemical similarity between Se and S, biochemical analogues are thought to be metabolized and transported via similar mechanisms. However, the exclusion of S was 1–2 orders of magnitude less pronounced than that of Se, suggesting Se was specifically excluded by these herbivores. The mechanism of this Se-specific exclusion may be that Se and S occur in different chemical forms, and only the Se form is expelled. Alternatively, Se and S may occur as analogs of the same form, and the Se-containing molecules are somehow differentiated by the transporter from their S analogs, e.g. by molecular weight or atomic size differences.

Investigation of the chemical forms of Se found that the three herbivores converted between 30–100% of the ingested C-Se-C forms into other organic and inorganic forms of Se, including SeCys, SeCystine, Se(GSH)_2_ and selenite. These forms are thought to be more toxic to insects than the ingested C-Se-C forms [18]. Apparently the overall levels of these individual Se-compounds in the insects were sufficiently low so as not to cause visible Se toxicity. In order to better elucidate this, further studies may focus on directly comparing different forms of Se and their relative toxicities to insects or their cell lines in growth media.

These results are of interest because they provide new evidence that Se hyperaccumulating plants live in symbiosis with a range of Se-resistant ecological partners, including these novel Se-excluding seed insect herbivores. In an earlier study by Freeman *et al*. [18] the mechanism of Se-resistance in a diamondback moth herbivore of *S. pinnata* was hypothesized to be the ability to keep ingested MeSeCys in its parent form, or rather the inability to demethylate it into the more toxic, potentially protein-accumulated form- SeCys. This may enable the Colorado population of diamondback moth to tolerate Se levels in its tissues up to 250 µg Se g^−1^ DW [18]. In all three seed herbivore insect species investigated in the current study, the apparent Se resistance mechanism appears to be the ability to actively exclude Se from bio-accumulating in their tissues and to excrete Se as a waste in frass. While the seed herbivores did metabolize the ingested MeSeCys, γ-glu-MeSeCys or selenocystathionine from the host plant seeds to relatively more toxic forms, they excluded these Se forms from their tissues, and in at least one case concentrated Se to much higher levels in frass. Our findings demonstrate the presence of a previously unreported Se-resistance mechanism for the ecological partners of these Se hyperaccumulator plants. Furthermore, the finding that these three seed insect herbivore species do not accumulate substantial Se levels has important implications for their potential to form a Se portal which could move Se up into higher trophic levels. Based on these data their relatively low Se concentrations are not expected to lead to any biomagnification of Se in their predators.

## Supporting Information

Figure S1 **Liquid Chromatography Mass Spectrometry (LC-MS) chromatograms from 50 mM HCL extracts of two replicate batches of** ***S. pinnata*** **seeds collected at Pine Ridge Natural Area, identifying the only detectable Se-compound as selenocystathionine (Mw 270 [M+H]).**
(TIF)

Material S1 **EDS spectra obtained from** ***A. bisulcatus*** **and** ***S. pinnata*** **seeds, as well as from seed chalcids and bruchid beetles that emerged from such seeds.**
(PDF)
