# Supplementary figures and images for: Selenium Hyperaccumulator Plants Stanleya pinnata and Astragalus bisulcatus Are Colonized by Se-Resistant, Se-Excluding Wasp and Beetle Seed Herbivores
Source: PLoS One. 2012 Dec 3;7(12):e50516. doi: 10.1371/journal.pone.0050516 (PMC3513300; doi:10.1371/journal.pone.0050516)

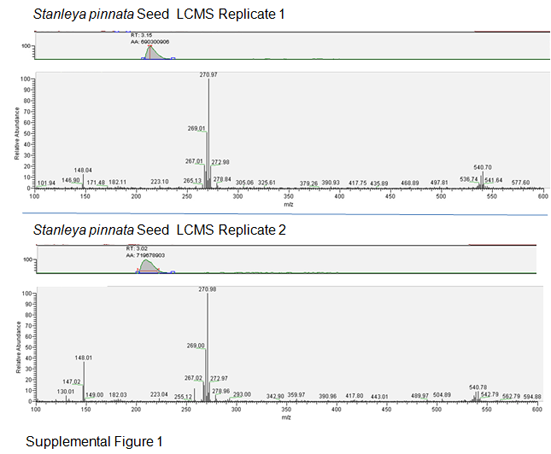

Supplement: Figure S1 — Liquid Chromatography Mass Spectrometry (LC-MS) chromatograms from 50 mM HCL extracts of two replicate batches of S. pinnata seeds collected at Pine Ridge Natural Area, identifying the only detectable Se-compound as selenocystathionine (Mw 270 [M+H]). (TIF) [file pone.0050516.s001.tif]
