# Supplementary material for: Selenium Hyperaccumulator Plants Stanleya pinnata and Astragalus bisulcatus Are Colonized by Se-Resistant, Se-Excluding Wasp and Beetle Seed Herbivores
Source: PLoS One. 2012 Dec 3;7(12):e50516. doi: 10.1371/journal.pone.0050516 (PMC3513300; doi:10.1371/journal.pone.0050516)
Supplement: Material S1 — EDS spectra obtained from A. bisulcatus and S. pinnata seeds, as well as from seed chalcids and bruchid beetles that emerged from such seeds. (PDF) [file pone.0050516.s002.pdf]

Project Selenium 23
Sample: Sample 3   Site of interest 1
ID: Astragalus bisulcatus mature seed (air dried, sectioned and Carbon coated)

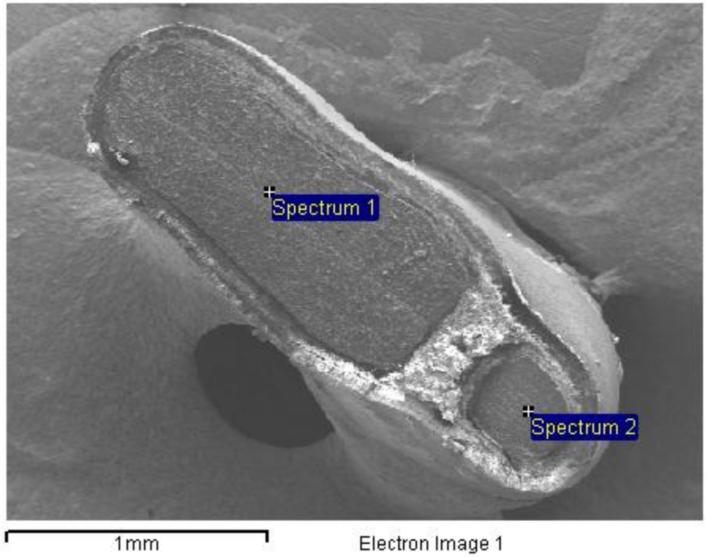

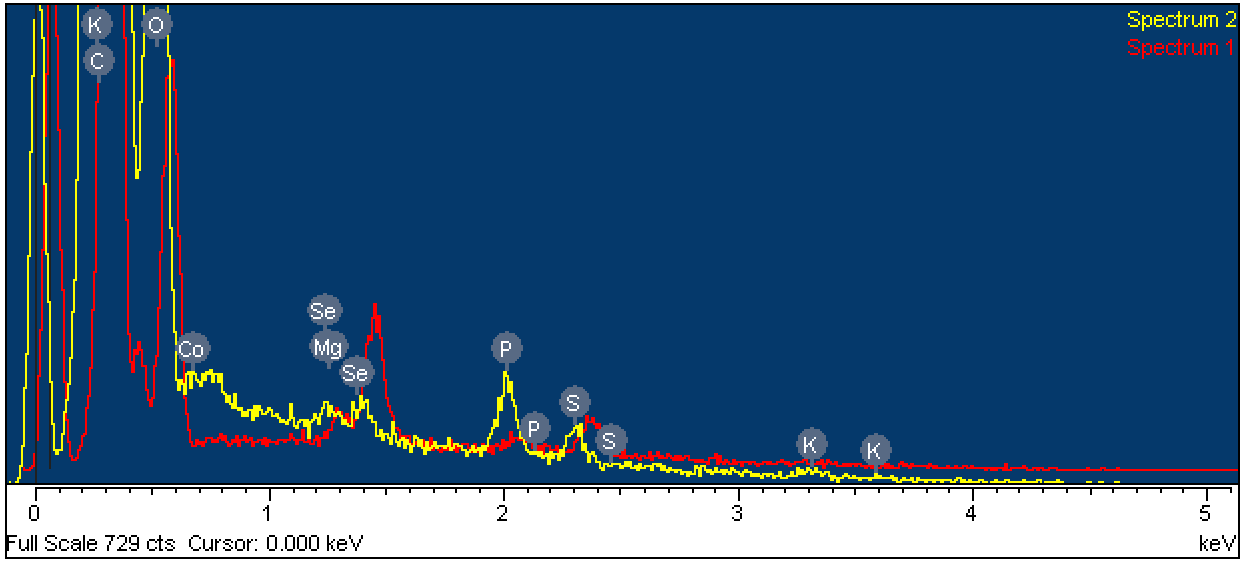

Processing option : All elements analysed (Normalised)

Spectrum	In stats.	O	Mg	P	S	K	Co	Se	Total
Spectrum 1	Yes	46.66	2.76	3.41	15.51			31.67	100.00
Spectrum 2	Yes	68.26	1.33	10.79	6.46	5.88	3.80	3.46	100.00
Max.		68.26	2.76	10.79	15.51	5.88	3.80	31.67	
Min.		46.66	1.33	3.41	6.46	0.00	0.00	3.46	

All results in weight%

Project Selenium 22
Sample: 3 Site of interest 7
ID: Astragalus bisulcatus mature seed. (air dried, sectioned and Carbon coated)

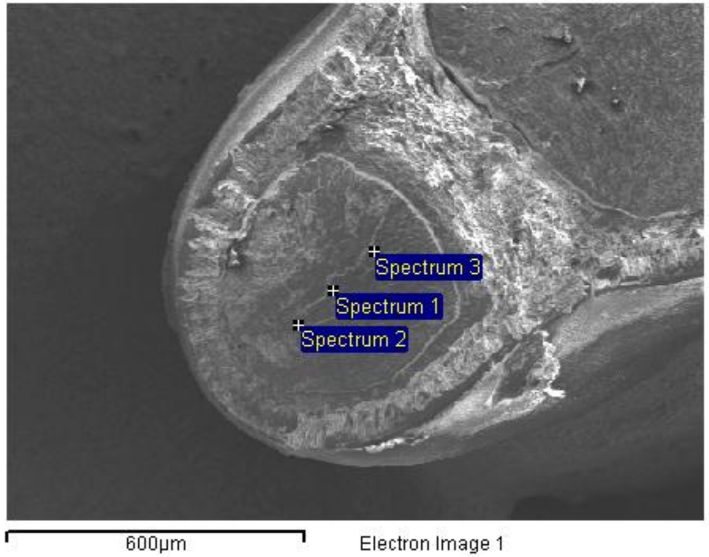

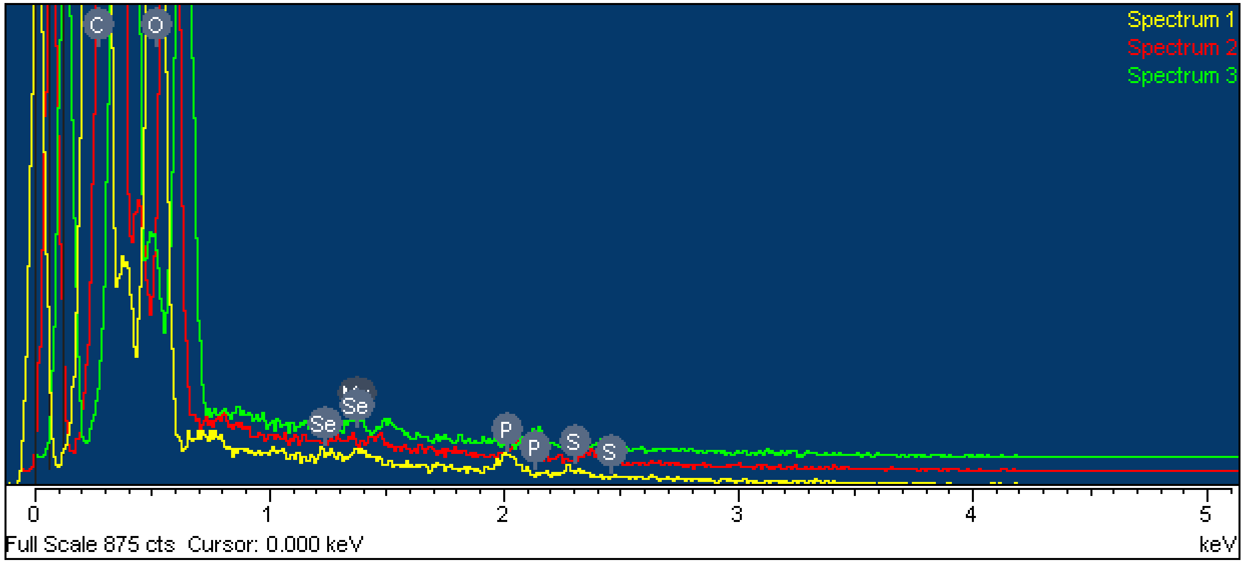

Processing option : All elements analysed (Normalised)

Spectrum	In stats.	O	Mg	P	S	Se	Total
Spectrum 1	Yes	89.26		5.86	2.72	2.17	100.00
Spectrum 2	Yes	88.38		5.69	3.52	2.41	100.00
Spectrum 3	Yes	83.25	2.09	6.38	3.29	4.98	100.00
Max.		89.26	2.09	6.38	3.52	4.98	
Min.		83.25	0.00	5.69	2.72	2.17	

All results in weight%

Project Selenium 22
Sample: 3 Site of interest 6
ID: Astragalus bisulcatus mature seed. (air dried, sectioned and Carbon coated)

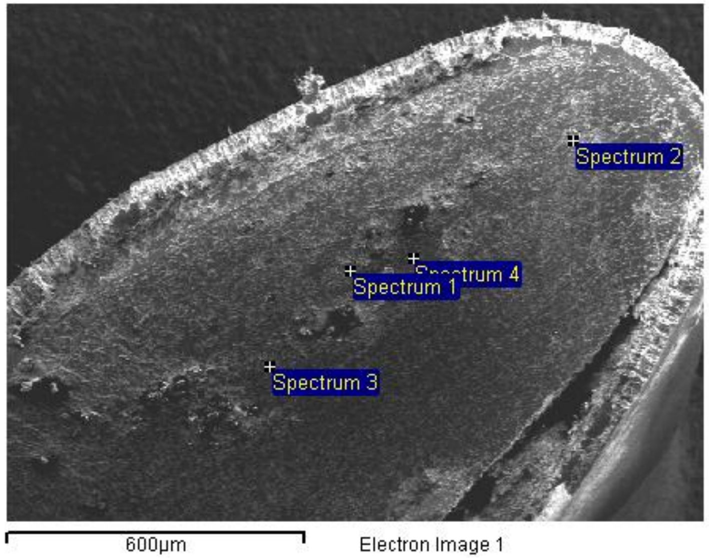

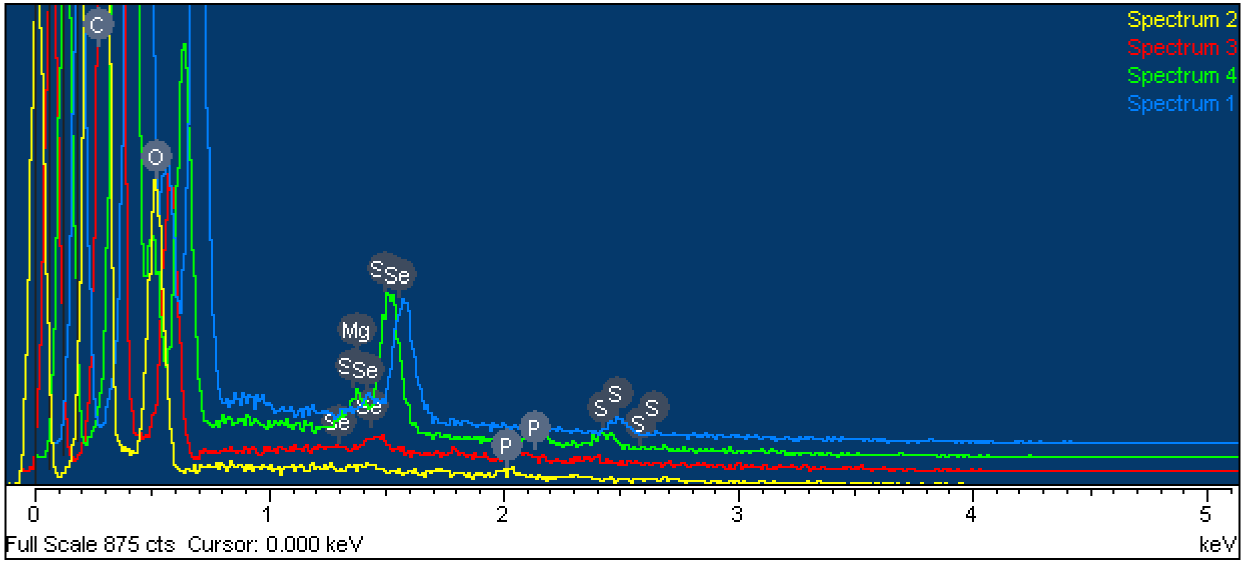

Processing option : All elements analysed (Normalised)

Spectrum	In stats.	O	Mg	P	S	Se	Total
Spectrum 1	Yes	66.12			6.17	27.71	100.00
Spectrum 2	Yes	92.69		7.31			100.00
Spectrum 3	Yes	83.61		6.18		10.21	100.00
Spectrum 4	Yes	46.13	4.12	5.62	6.30	37.83	100.00
Max.		92.69	4.12	7.31	6.30	37.83	
Min.		46.13	0.00	0.00	0.00	0.00	

All results in weight%

Project Selenium 24
Sample: Sample 3 (green seed) Site of interest 1
ID: Astragalus bisulcatus green seed (cryo stage fractured Au coated)

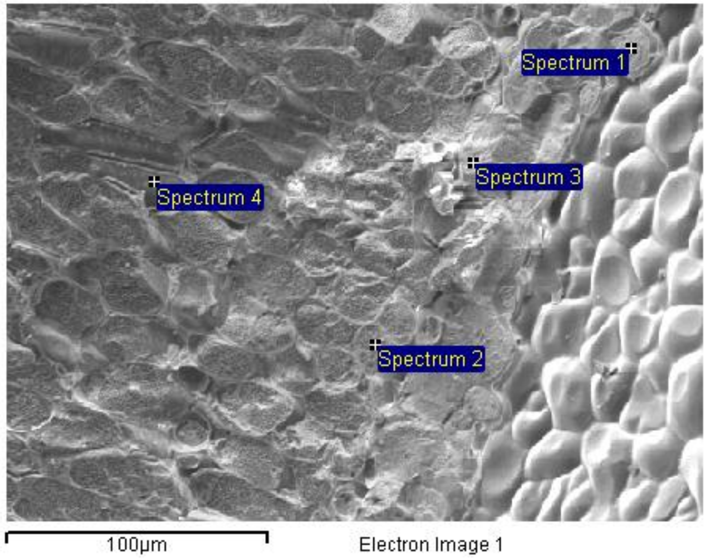

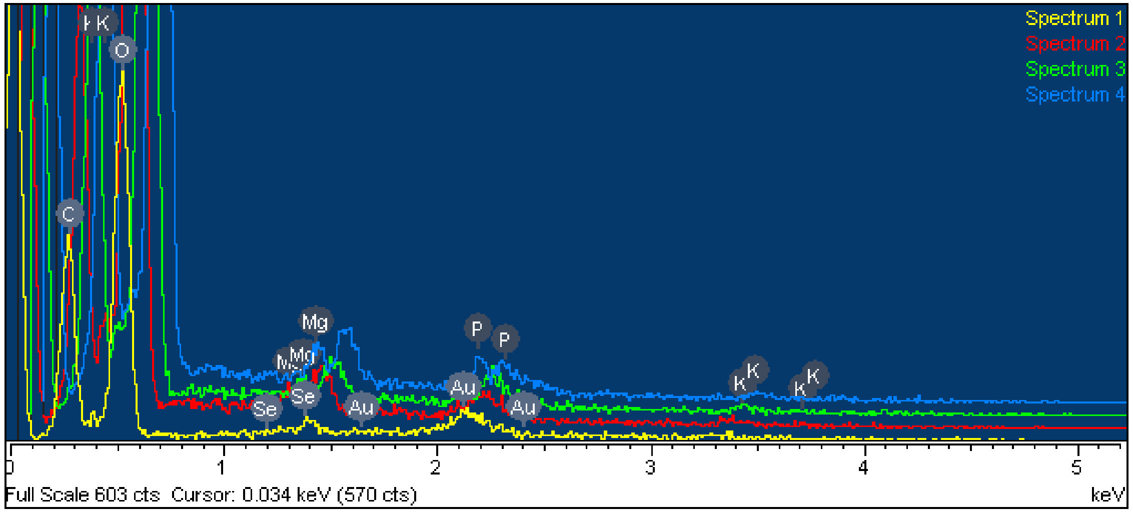

Processing option : All elements analysed (Normalised)

Spectrum	In stats.	C	O	Mg	P	K	Se	Total
Spectrum 1	Yes	32.97	64.25				2.78	100.00
Spectrum 2	Yes	17.13	79.43	0.50			2.94	100.00
Spectrum 3	Yes	23.87	68.72	0.43		4.39	2.59	100.00
Spectrum 4	Yes	22.01	69.43	0.88	1.59	2.96	3.13	100.00
Max.		32.97	79.43	0.88	1.59	4.39	3.13	
Min.		17.13	64.25	0.00	0.00	0.00	2.59	

All results in weight%

Project Selenium 24
Sample 3 SOI 3 Astragalus bis. green seed. Cryo Fractured seed case in and Au coated

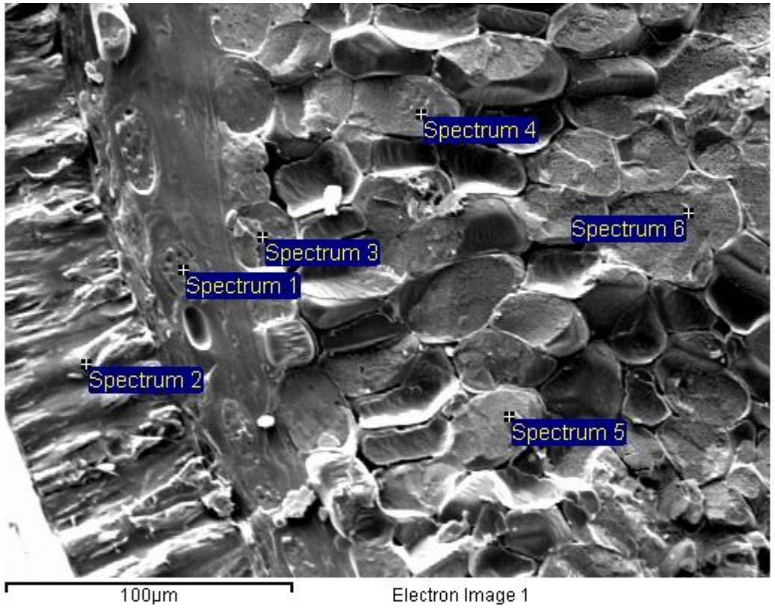

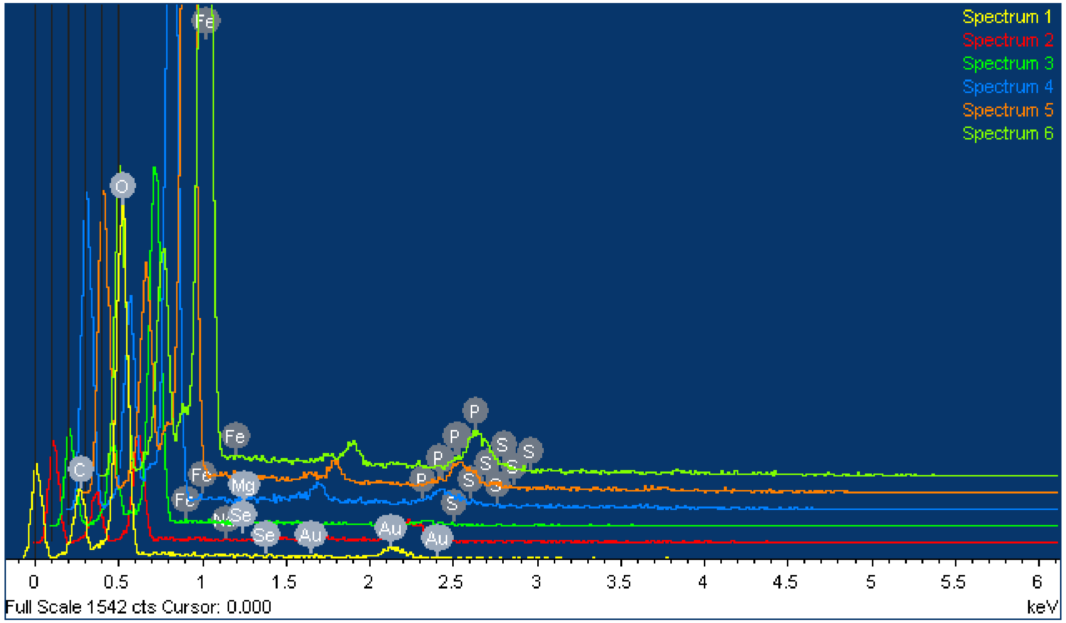

Processing option : All elements analysed (Normalised)

Spectrum	In stats.	C	O	Na	Mg	P	S	Fe	Se	Total
Spectrum 1	Yes	16.75	82.55		0.18				0.52	100.00
Spectrum 2	Yes	30.55	67.61	0.46					1.38	100.00
Spectrum 3	Yes	17.22	80.65				0.43	1.24	0.45	100.00
Spectrum 4	Yes	20.38	74.61		0.33	0.32	0.73	0.96	2.66	100.00
Spectrum 5	Yes	15.30	80.56		0.36	0.50	0.44	0.48	2.36	100.00
Spectrum 6	Yes	17.23	76.88		0.48	0.88	0.82	0.87	2.84	100.00
Max.		30.55	82.55	0.46	0.48	0.88	0.82	1.24	2.84	
Min.		15.30	67.61	0.46	0.18	0.32	0.43	0.48	0.45	

All results in weight%

Project Selenium 25
Sample: Sample 6 Site of interest 3 fractured surface of the seed testa
ID: Astragalus bisulcatus fruit pods and seed (cryo stage fractured Au coated)

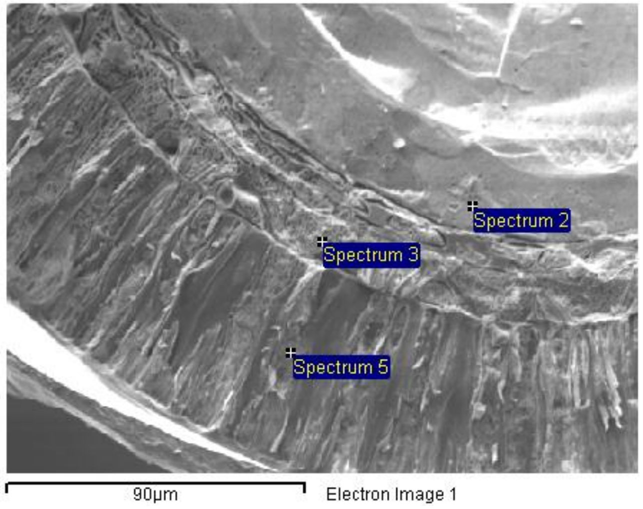

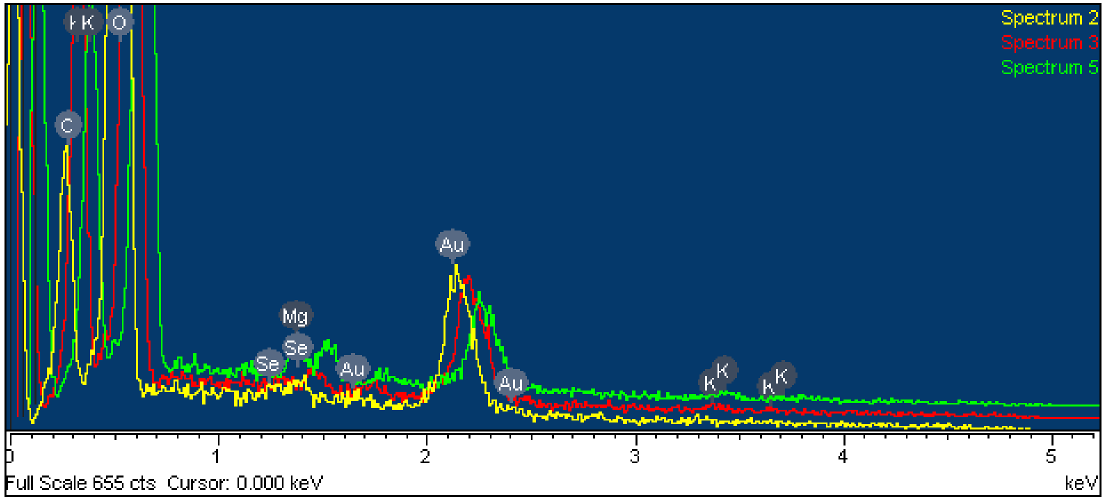

Processing option : All elements analysed (Normalised)

Spectrum	In stats.	C	O	Mg	K	Fe	Se	Total
Sum Spectrum	Yes	30.44	67.07	0.26		1.60	0.63	100.00
Spectrum 2	Yes	10.54	88.44				1.02	100.00
Spectrum 3	Yes	22.25	72.93		3.62		1.21	100.00
Spectrum 4	Yes	21.12	77.82				1.05	100.00
Spectrum 5	Yes	16.69	75.42	1.32	4.00		2.57	100.00
Max.		30.44	88.44	1.32	4.00	1.60	2.57	
Min.		10.54	67.07	0.00	0.00	0.00	0.63	

All results in weight%

Project Selenium 23
Sample 4 SOI 1 Stanleya pinnata mature seed. Air dried sectioned and Carbon coated

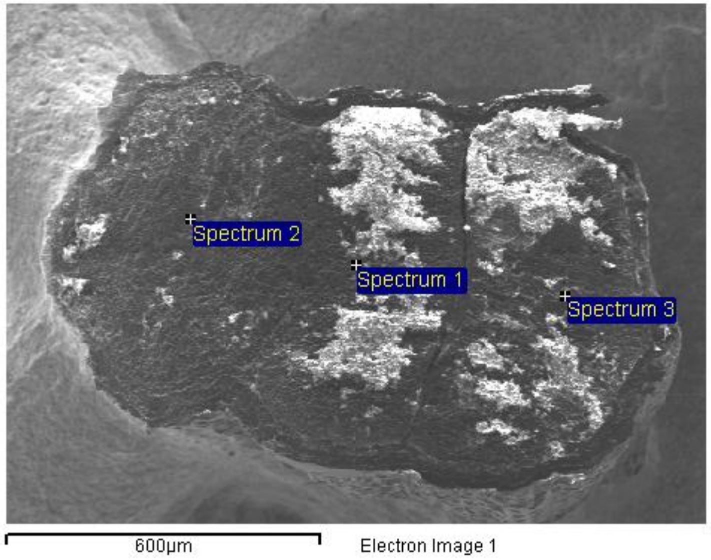

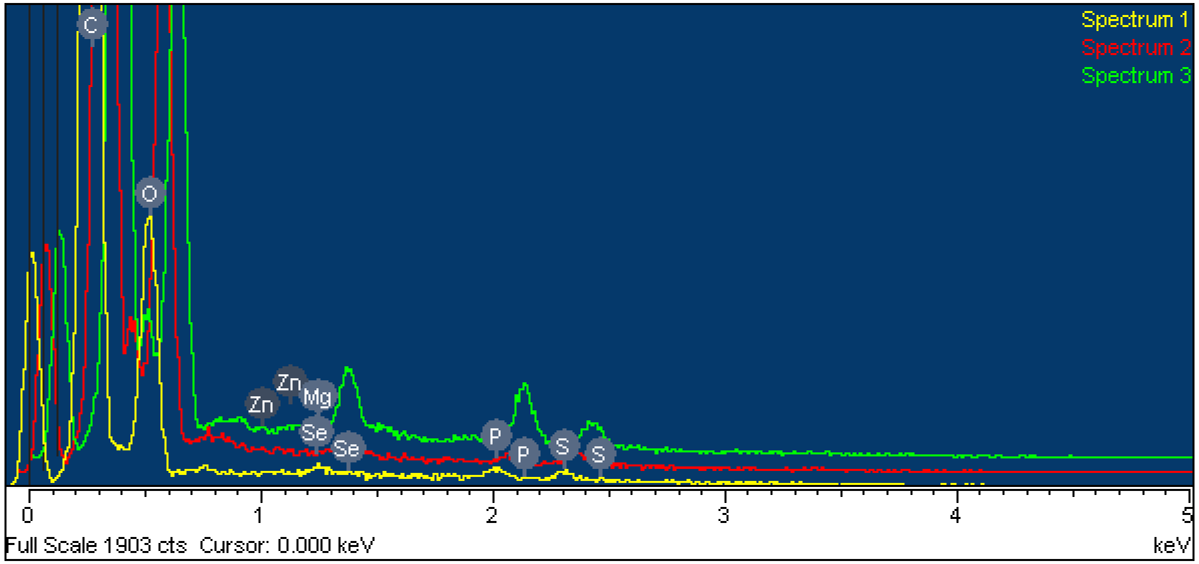

Processing option : All elements analysed (Normalised)

Spectrum	In stats.	O	Mg	P	S	Zn	Se	Total
Spectrum 1	Yes	78.63	2.26	7.35	9.40		2.36	100.00
Spectrum 2	Yes	87.67	1.00	3.75	6.79		0.79	100.00
Spectrum 3	Yes	61.35	7.62	17.40	10.39	1.36	1.89	100.00
Max.		87.67	7.62	17.40	10.39	1.36	2.36	
Min.		61.35	1.00	3.75	6.79	0.00	0.79	

All results in weight%

Project Selenium 25
Sample 5 SOI 2 Stanleya pinnata green seed. Cryo coated Au

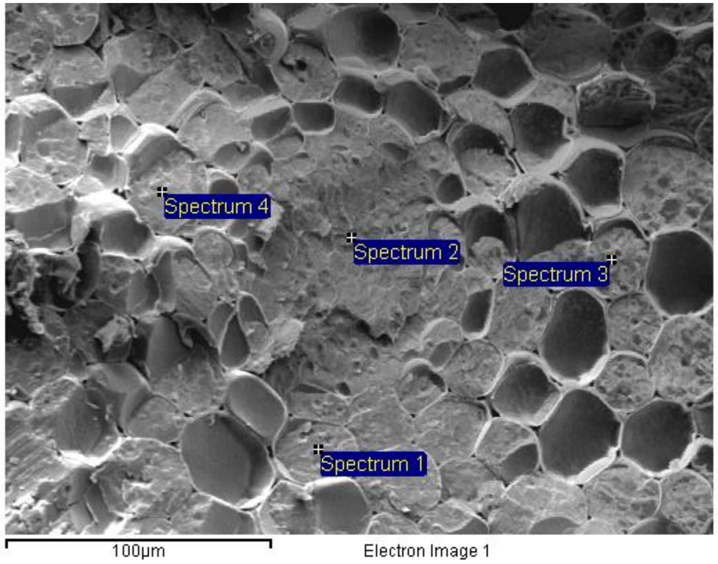

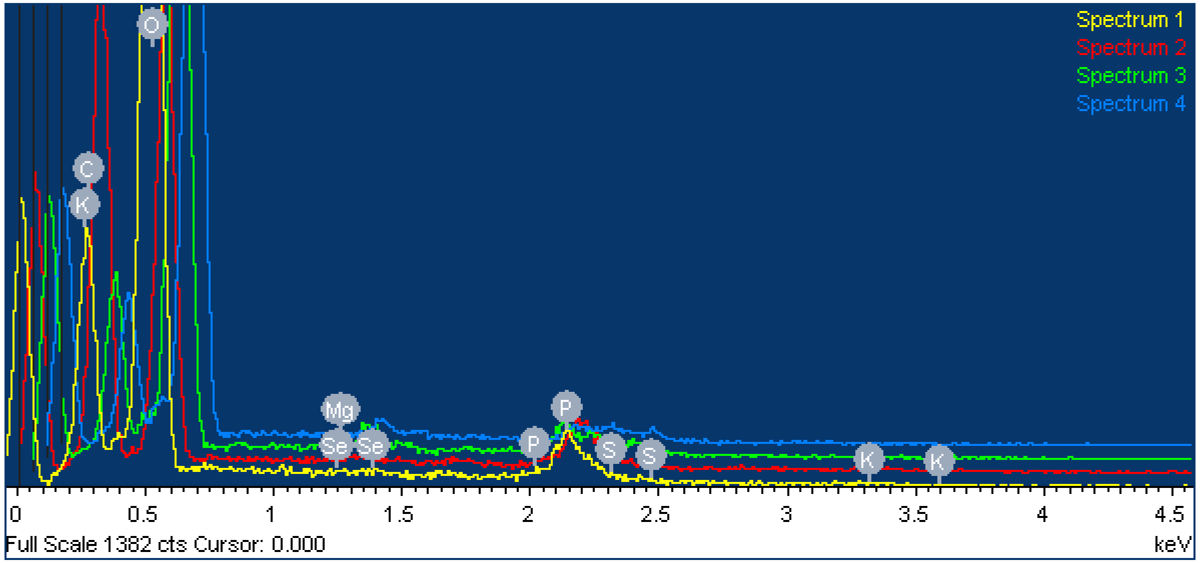

Processing option : All elements analysed (Normalised)

Spectrum	In stats.	C	O	Mg	P	S	K	Se	Total
Spectrum 1	Yes	16.13	80.12	0.09	0.40	1.08	1.71	0.46	100.00
Spectrum 2	Yes	44.66	49.79	0.19	0.76	1.44	3.05	0.12	100.00
Spectrum 3	Yes	15.00	75.71	1.48	4.26	2.41	0.26	0.88	100.00
Spectrum 4	Yes	11.80	80.61	1.10	2.32	2.57	0.79	0.80	100.00
Mean		21.90	71.56	0.72	1.93	1.88	1.45	0.56	100.00
Std. deviation		15.28	14.68	0.68	1.76	0.73	1.22	0.35	
Max.		44.66	80.61	1.48	4.26	2.57	3.05	0.88	
Min.		11.80	49.79	0.09	0.40	1.08	0.26	0.12	

All results in weight%

Comment:Bruchid beetle adult abdomen posterior Cryo planed Au coated

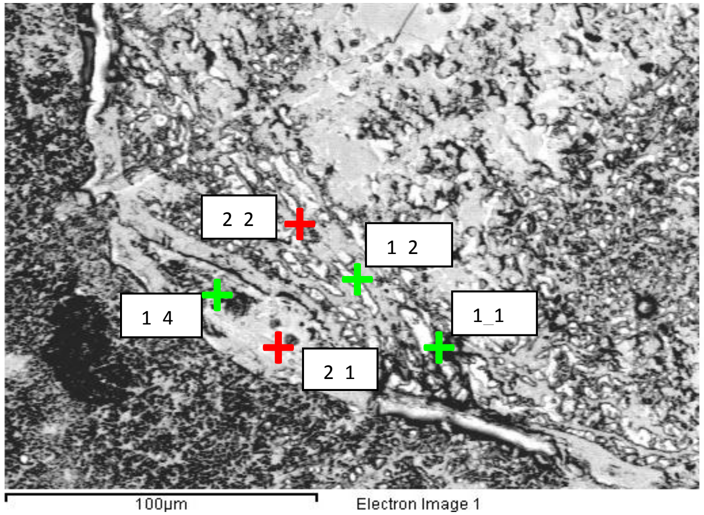

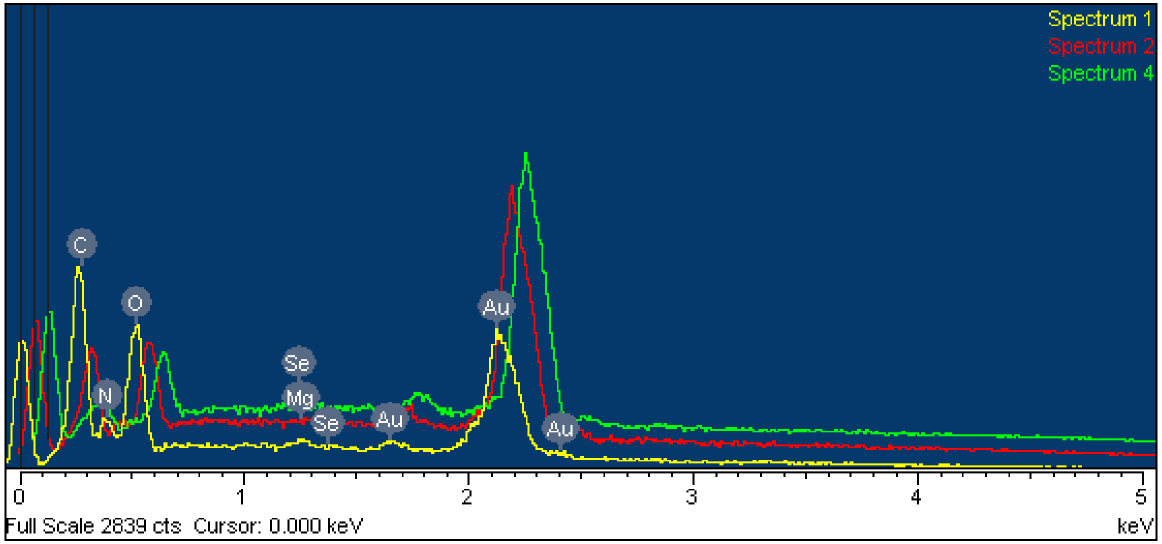

Processing option : All elements analysed (Normalised)

Spectrum	In stats.	C	O	Mg	P	S	Se	Total
Spectrum 1	Yes	44.84	36.59	1.91	10.59	4.91	1.15	100.00
Spectrum 2	Yes	38.19	57.75	2.20			1.86	100.00
Spectrum 4	Yes	18.16	79.36	0.82			1.66	100.00
Spectrum 1	Yes	62.83	18,66	1.50		15.89	1.12	100.00
Spectrum 2	Yes	48.86	44.00	1.93		3.77	1.45	100.00
Max		62.83	79.36	1.93	10.59	15.89	1.86	
Min.		18.16	18.66	0.82	10.59	3.77	1.12	

All results in weight%
